# Increased Access to Antiretroviral Therapy Is Associated with Reduced Maternal Mortality in Johannesburg, South Africa: An Audit from 2003-2012

**DOI:** 10.1371/journal.pone.0168199

**Published:** 2016-12-29

**Authors:** Vivian Black, Andrew D. Black, Helen V. Rees, Franco Guidozzi, Fiona Scorgie, Matthew F. Chersich

**Affiliations:** 1 Wits Reproductive Health and HIV Institute, Faculty of Health Sciences, University of the Witwatersrand, Johannesburg, South Africa; 2 Clinical Microbiology and Infectious Diseases, Faculty of Health Sciences, University of the Witwatersrand, Johannesburg, South Africa; 3 Department of Obstetrics and Gynaecology, Faculty of Health Sciences, University of the Witwatersrand, Johannesburg, South Africa; University of Missouri Columbia, UNITED STATES

## Abstract

**Objective:**

To assess the impact of expanded access to antiretroviral treatment (ART) on maternal mortality in Johannesburg, South Africa between 2003 and 2012.

**Methods:**

Audit of patient files, birth registers and death certificates at a tertiary level referral hospital. Cause of death was assigned independently, by two reviewers. We compared causes of deaths and the maternal mortality ratios (MMR, deaths/100,000 live births) over three periods corresponding to changes in government policy on ART provision: period one, 2003–2004 (pre-ART); period two, 2005–2009 (ART eligibility with CD4 count <200cells/μL or WHO stage 4 disease); and period three, 2010–2012 (eligibility with CD4 count <350 cells/μL).

**Results:**

There were 232 deaths and 80,376 deliveries in the three periods. The proportion of pregnant women tested for HIV rose from 43.4% in 2003 to 94.6% in 2012. MMR was 301, 327 and 232 in the three periods, (p = 0.10). The third period MMR was lower than the first and second combined (p = 0.03). Among HIV-positive women, the MMR fell from 836 in the first time period to 431 in the third (p = 0.008) but among HIV negative women it remained unchanged over the three periods, averaging 148. Even in the third period, however, the MMR among HIV-infected women was 3-fold higher than in other women. Mortality from direct obstetric causes such as hemorrhage did not decline over time, but deaths from tuberculosis and HIV-associated malignancy did. In 38.3% of deaths, women had not attended antenatal care.

**Conclusion:**

Higher coverage of HIV testing and ART has substantially reduced MMR in this hospital setting. Though the gap in MMR between women with and without HIV narrowed, a third of deaths still remain attributable to HIV. Lowering overall MMR will require further strengthening of HIV services, increased antenatal care coverage, and improved care for obstetric emergencies at all levels of care.

## Introduction

Over the past fifteen years,[1} there has been a substantial global decline in maternal mortality. The decline has been much slower in sub-Saharan Africa, largely due to the HIV epidemic in this region.[1–3] In 2013, the maternal mortality per 100,000 lives births was 280 in southern Africa, compared to 209 globally and 12 in high-income countries. [1] These differing levels reflect the varying ability of countries to address both the clinical causes of maternal deaths, including HIV, as well as the underlying social and economic determinants of maternal death. Narrowing the gap in maternal mortality across regions requires a thorough understanding of the causes of maternal deaths, supported by interventions that address avoidable factors in high-burden settings.[4] Investigating the contribution of antiretroviral treatment (ART) to mitigating these gaps is especially important in areas with a high HIV burden.

HIV infection impacts on women’s health in many ways during pregnancy. HIV-infected women have higher risks of both indirect maternal deaths (such as from tuberculosis), and of deaths from direct obstetric causes[5] HIV disease generates a huge demand for care in countries such as South Africa, where HIV prevalence in pregnant women has remained largely unchanged over the past decade (between 29.1% and 30.2% in the years from 2004 to 2012).[6] Access to ART has improved considerably in South Africa in recent years, with an estimated 3.38 million people receiving ART by 2015.[7] Prevention of mother-to-child transmission (PMTCT) programs now reduce the risk for transmission of HIV to infants to under three percent [8] This considerable demand for services may divert resources away from obstetric care and detract from initiatives to enhance the quality of other maternal health services.[9] Equally, there are potential synergies between ART scale-up and health service productivity, which could mitigate any negative consequences that might arise from providing the volume of HIV interventions required.

Given the complexity of interactions between HIV and maternal health services, it is important to document the impact of progressively increasing access to ART on the maternal mortality of HIV-infected compared to HIV-uninfected women. At the same time, it is important to monitor trends in direct obstetric deaths over time, as these provide a measure of the quality of routine and emergency obstetric care. The study reported here reviews patterns of maternal mortality, together with changes in the causes of maternal deaths at a large tertiary-level facility in Johannesburg, Gauteng Province, South Africa. Three time periods are compared: 2003–2004, 2005–2009 and 2010–2012, each of which correspond to shifts in government policy on provision of ART.

## Materials and Methods

### Study setting

Charlotte Maxeke Johannesburg Academic Hospital (CMJAH) provides tertiary-level services for patients in a densely populated sub-district of greater Johannesburg. High-risk women from the surrounding seventeen primary and one secondary facilities are referred to the hospital during pregnancy or childbirth. However, deliveries are not confined to high risk women as only two other smaller facilities provide labour and delivery services and the majority of women in the district have their deliveries at CMJAH. About 95% of pregnant women in the province attend antenatal care at least once, and even more have a trained nurse, midwife, or doctor present at birth.[10] At labor onset, women present for assessment and admission to CMJAH’s 100-bed maternity unit. Provided a woman is well, she is usually discharged within 24 hours of childbirth, or after three days following complicated deliveries or Cesarean sections. Women are asked to attend their local clinic for postpartum assessment within a week of delivery and to continue HIV care if they are HIV positive, but uptake of these services is low.[8]

### HIV services and antiretroviral eligibility

HIV testing in South Africa was initially based on voluntary counselling and testing, but this changed to provider-initiated HIV testing and counselling (PITC) in 2008. For pregnant women, HIV testing occurred at the first visit for antenatal care and was repeated at the 32^nd^ week of pregnancy in women who initially tested negative. If HIV testing was declined by a pregnant woman, it was offered again at subsequent visits and during labor.

**Fig 1:**
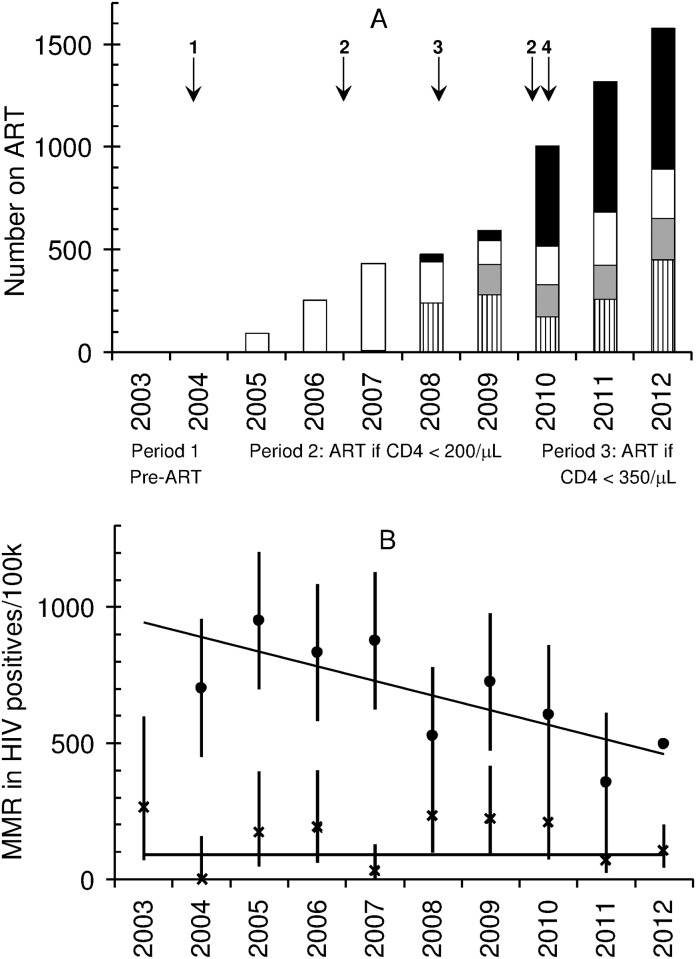
A Number of pregnant women initiated on ART in a sub-district of Gauteng Province, South Africa from 2004 to 2012, and key events. Key: vertical stripes = ART initiation at primary health care antenatal clinic (ANC); grey shading = ART initiation at district hospital ANC: white = ART initiation at Charlotte Maxeke Johannesburg Academic Hospital ANC; black = women who had initiated ART prior to pregnancy. Key events: 1 = ART initiation for people with a CD4 count < 200cells/μL or WHO stage 4, 2 = Labour dispute in health sector, 3 = Decentralisation of ART provision to primary care facilities, 4 = ART initiation for pregnant women if their CD4 count < 350 cells/μL. B Maternal mortality rate among women from 2004 to 2012 at Charlotte Maxeke Johannesburg Academic Hospital. Circle = HIV-positive women MMR; diamond = HIV-negative women. Weighted least-squares fit of linear association between year and number of deaths. The slope of the line for HIV-positive women is -54.4/year, 95%CI = -90.4 to -18.4; p = 0.007. The slope of the line for HIV-negative women is 0.08/year, 95%CI = -23.1–23.3; p = 0.994

As indicated above, ART provision is characterized by three distinct time periods. In the first, 2003 to March 2004, ART was not available in the state sector, and HIV-infected women only received single-dose nevirapine for PMTCT prophylaxis, which was the prevailing PMTCT regimen until 2008, when zidovudine maternal and infant prophylaxis were introduced. In the second period, between April 2004 and March 2010, HIV-infected people (including pregnant women) with a CD4 count ≤200 cells/μL or WHO stage IV clinical disease, qualified for ART and were usually initiated on stavudine, lamivudine and nevirapine. Efavirenz was included as an alternative to nevirapine after the first trimester of pregnancy. Due to delays in implementing the ART guidelines for pregnant women at CMJAH, very few pregnant women were initiated on ART in 2004 “Fig 1”.[11] For this audit, therefore, the months April to December 2004 are considered part of period one. The third period begins from April 2010, when the CD4 cell count threshold for ART initiation was raised. Pregnant women with a CD4 count ≤350 cells/μL were offered ART, consisting of nevirapine, lamivudine and tenofovir. Because of concerns about teratogenicity at the time, efavirenz was no longer offered to pregnant women, barring clinical conditions that contraindicated the use of both nevirapine and lopinovir/ritonovir. However, following reports of maternal deaths related to nevirapine toxicity, the guidelines were reversed in mid-2012, with efavirenz replacing nevirapine in ART regimens.[12] As data on number of births were only available for 2010 and not for individual months, the months January to March 2010 was included in the third period, even though women with a CD4 count between 200 cells/μL and 350 cells/μL did not initiate ART during those three months. Cotrimoxazole prophylaxis was provided to HIV-infected pregnant women throughout the study period, as was isoniazid prophylaxis for women with no suspicion of tuberculosis.

Some aspects of service delivery warrant description. Firstly, in the second and third periods, women testing HIV positive had a CD4 cell count test and results were available two weeks later, as point of care tests were not available at the time.[11] Secondly, HIV testing and other services were affected for extended periods in 2007 and 2010 by a series of labor disputes, involving nurses, and by interruptions in payments for lay counselors.[13–15] Finally, in early 2008, ART was provided only at CMJAH and two large referral facilities, but by the end of 2012 the services had expanded progressively to cover all 17 facilities in the sub-district. Prior to 2008, women attending facilities where ART was not available were referred to CMJAH antenatal clinic if eligible for treatment.

### Data extraction and analysis

CMJAH patient and laboratory records were audited for all facility-based maternal deaths between 2003 and 2012, and data extracted on the characteristics of women who had died, their HIV status, obstetric history, clinical condition and timing of death in relation to pregnancy. Maternal deaths were defined as the death of a woman at the facility during pregnancy or within 42 days of childbirth.[16] Information was not available about maternal deaths that occurred at home or at other facilities.

The cause of death in each case was assigned following discussions at mortality meetings of the CMJAH Department of Obstetrics and Gynaecology. An internal medicine specialist (AB) and an infectious diseases specialist (VB), using all available evidence including clinical records, prescription charts, nursing reports, laboratory results and mortality audit reports, then independently verified cause of death and its classification. Any discrepancies between reviewers were resolved through discussion. Women who had died following a spontaneous or induced abortion (termination of pregnancy before 20 weeks gestation)[17] were considered as having died during pregnancy. Deaths were classified as direct obstetric deaths (due to obstetric complications including pregnancy hypertensive disorders, hemorrhage, pulmonary embolism, pregnancy related infections and iatrogenic factors), indirect obstetric deaths (resulting from a pre-existing disease that was aggravated by the physiological effects of pregnancy, such as cardiac disease, end organ disease and non-pregnancy related infections), or unknown.[18] Deaths that were considered accidental or incidental were excluded.

The total number of deliveries and HIV status of women was determined through a review of hospital birth registers. The District Health Information System (DHIS) provided information on the total number of women in the sub-district who had initiated ART or were already receiving treatment at the time of pregnancy (over two thirds of the women in the sub-district give birth at CMJAH).

The annual maternal mortality ratio (MMR = number of maternal deaths at CMJAH per 100,000 live births at CMJAH) was calculated for the facility, and for women with or without HIV infection, or of unknown HIV status. The proportional reduction in MMR that would occur if no women had HIV was estimated by the population attributable fraction (PAF). This was calculated as PAF = [p(r_1_-r_0_)]/r where r_1_ is MMR in HIV positive women, r_0_ is MMR in HIV negative women, r is the overall MMR and p is prevalence of HIV.

Data were analyzed using Intercooled Stata version 12.0 (Stata-Corp, LP, College Station, TX). Patterns in maternal deaths and in the performance of the HIV programme were compared between the three time periods. Chi-square tests were used to assess differences between categorical variables. A Chi-square test for trend determined whether there was a trend over the three periods in the proportions within each exposure category, together with a chi-squared test of homogeneity of odds was also done.[19] Approval for the study was given by the Human Research Ethics Committee of the University of the Witwatersrand (M101150).

## Results

### Study population and service coverage

Over the full ten years, a total of 236 maternal deaths occurred at the facility, four were considered incidental (a drowning, an herbal intoxication, a suicide and a motor vehicle accident) and were excluded from analysis, leaving 232 deaths that were included in the analysis. During the same time period there were 80, 376 live births, generating an overall MMR of 288.6 deaths/100,000 live births). Considerably more births took place in the third period (9487.7/year) than in the preceding years (about 7400/year in periods one and two). Aside from 2010, the year of the labor disputes, HIV testing increased year on year from 43.4% in 2003 to 94.6% in 2012 (9272/9802, *P*<0.001) “Table 1”. Among women delivering at the facility who had a known HIV status, the HIV prevalence declined with each study period, from 39.4% in 2003–2004 to 31.5% in 2010–2012 (*P*<0.001)”Table 1”.

**Table 1:** Facility-based maternal mortality ratios (deaths/100 000 live births) over ten years at Charlotte Maxeke Johannesburg Academic Hospital, South Africa, by HIV status.

Var. cat	Variable	2003–2004	2005–2009	2010–2012	*P*^+^
**HIV services**	**HIV prevalence**^&^ % (n/N)	39.4 (2751/6978)	34.9 (9283/26,636)	31.5 (7411/23,546)	<0.001
	**Testing coverage** % (n/N)	46.7 (6978/14935)	72.0 26,636/36,978)	82.7 (23,546/28,463)	<0.001
**MMR for key causes of death** (n deaths; N births)	**All causes of direct obstetric deaths**	127.2 (19; 14,935)	167.7 (62; 36,978)	147.6 (42; 28,463)	1.0
	**Hypertension**	73.7 (11;14,935)	48.7 (18; 36,978)	52.7 (15; 28,463)	0.64
	**Obstetric hemorrhage**	26.8 (4; 14,935)	35.2 (13; 36,978)	31.6 (9; 28,463)	0.96
	**Pregnancy-related sepsis**	26.8 (4; 14,935)	56.8 (21; 36,978)	42.2 (12; 28,463)	0.99
	**All causes of indirect obstetric deaths**	133.9 (20; 14,935)	146.0 (54; 36,978)	84.3 (24; 28,463)	0.04
	**Non pregnancy-related infection**	100.4 (15; 14,935)	105.5 (39; 36,978)	52.7 (15; 28,463)	0.03
	**Tuberculosis**	53.6 (8; 14,935)	40.6 (15; 36,978)	7.0 (2; 28,463)	0.003
**MMR in sub-groups and overall** (n deaths; N births)	**HIV-positive women**	836.1 (23; 2751)	711.0 (66; 9283)	431.2 (32; 7411)	0.008
	**HIV-negative women**	118.3 (5; 4227)	167.1 (29; 17,353)	136.4 (22; 16,136)	0.89
	**Women unknown status**	213.6 (17; 7957)	251.4 (26; 10,342)	244.1 (12; 4917)	0.69
	**OVERALL MMR** 95%CI	301.3 (45; 14,935) 219.9–403.0	327.2 (121; 36978) 271.6–390.9	231.9 (66; 28,463) 179.4–294.9	0.10

MMR maternal mortality ratio (number of maternal deaths per 100 000 live births).

^&^Prevalence among those with known HIV status.

^+^Chi-square test for trend. Non pregnancy related infection includes tuberculosis, pneumonia sepsis, meningitis and encephalitis

Data from the whole sub-district shows that the number of pregnant women receiving ART increased annually, from 255 in 2005, to 2017 in 2012 “Fig 1”. Much of this rise can be ascribed to the large numbers of women initiating ART at primary health clinics from 2008 onwards, following policy initiatives to decentralize these services. By 2012, 45.3% of women in the sub-district who were taking ART during pregnancy had already initiated treatment prior to pregnancy (914/2017).

### Changes in maternal mortality ratio over time

The overall facility-based MMR in the third period was 231.9, 0.72 fold lower than the MMR in the first and second periods (95%CI OR = 0.54–0.96; P = 0.03). This reduction is attributable to changes in MMR among HIV-infected women, which were: 836.1 in period one; 711.0 in period two; and 431.8 (95% CI = 295.5–609.0) in period three (*P* = 0.008).

Despite reductions in deaths among HIV-infected women, the MMR in the third period was still 3.2 fold higher among HIV-positive than negative women, although it was 4.1 fold higher in the second period and 7.1 times higher in the first period. Even in the third period, with the increased availability of ART, 33.3% of maternal deaths can be attributed to HIV, down from about 44.1% in the first period and 41.9% in the second.

No changes over time were noted in the MMR from direct obstetric causes of deaths “Table 1”. With indirect causes, however, in the third period, the MMR was 84.3, lower than in the preceding periods (133.9 in period 1 and 146.0 in period 2; p = 0.04). A stepwise reduction in MMR from tuberculosis was noted: period 1 = 53.6, period 2 = 40.6 and period 3 = 7.0 (p = 0.003).

### Characteristics and causes of maternal deaths

The median age of the women who died was 29 years (IQR = 25.5–33.5), with no difference detected over time, or between HIV negative and positive women “S1 Table”. Median gravidity was 3 in HIV-negative women (IQR = 2–4), and 2 in HIV-positive women (IQR = 2–3; *P* = 0.95). Most of the HIV-infected women who died had advanced HIV disease, with similar CD4 cell counts across the time periods (median 71 cells/μL; interquartile range = 21–203 cells/μL) and 58.1% had WHO clinical stage 4 disease. Among HIV-infected women who died, none were receiving ART in the first period, whereas 20.5% were on ART in the second period (9/44) and 28.6% in the third (8/28; *P* = 0.39). Half the HIV-positive women had a history of tuberculosis (41/79), compared to none of the negative women (*P*<0.001).

**Fig 2:**
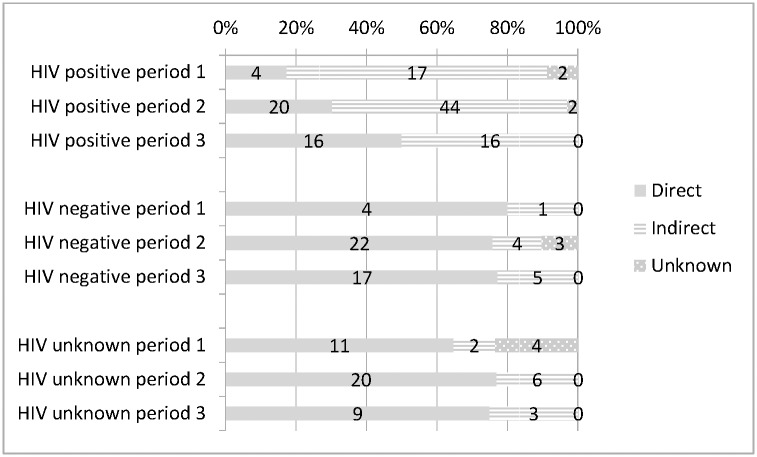
Proportion of deaths from direct and indirect obstetric causes by HIV status and time period.

In each time period, about 80% of deaths among HIV-negative women were due to direct obstetric causes “Fig 2”. By contrast, the proportion of deaths from direct causes in HIV-positive women was 17% in the first period, and rose 2.08 fold with each time period (95%CI OR = 1.19–3.65; *P* = 0.01).

Overall, tuberculosis was responsible for 17.8% of deaths in the first period (8/45), 12.4% in the second (15/121) and 3.0% in the third (2/66); a 54% reduction in the odds of death from tuberculosis with each period: (95%CI 25%-83%; *P* = 0.01) “Table 2”. Malignancy was the cause of death in 3 of the 45 women who died in period one (all Kaposi’s sarcoma), while of the 187 women who died in periods two and three, only one had lymphoma and another breast cancer (6.7% in period one versus 1.1% in period two and three; *P* = 0.02). Two deaths in the second time period were ascribed to adverse effects of ART: one to lactic acidosis due to stavudine, and the other to development of toxic epidermal necrolysis following nevirapine-based treatment. Across time periods, no change was detected in the proportion of women who died following an abortion (15.5% overall, 31/200).

**Table 2:** Causes of maternal deaths at Charlotte Maxeke Johannesburg Academic Hospital, comparing the periods pre-ART, ART initiation at CD4 <200 and ART starting at CD4 <350.

Condition	Period one: pre-ART 2003–2004 (45 deaths)					Period two: ART initiation at CD4 <200 2005–2009 (121 deaths)					Period three: ART initiation at CD4 <350 2010–2012 (66 deaths)					*P
	Death category N	HIV positive N = 23	HIV negative N = 5	HIV unknown N = 17	Total N	Death category N	HIV positive N = 66	HIV negative N = 29	HIV unknown N = 26	Total N	Death category N	HIV positive N = 32	HIV negative N = 22	HIV unknown N = 12	Total N	
Hypertensive disorders	**Direct 19**	1	2	8	**11**	**Direct 62**	3	5	10	**18**	**Direct 42**	3	8	4	**15**	0.997
Obstetric hemorrhage		2	1	1	**4**		2	6	5	**13**		5	3	1	**9**	0.422
Iatrogenic		0	0	0	**0**		1	1	0	**2**		0	0	0	**0**	0.852
Pulmonary embolism		0	0	0	**0**		3	4	0	**7**		3	2	0	**5**	0.091
Pregnancy-related sepsis		1	1	2	**4**		11	6	4	**21**		5	3	4	**12**	0.225
Other		0	0	0	**0**		0	0	1	**1**		0	1	0	**1**	0.397
Cardiac	**Indirect 20**	1	1	0	**2**	**Indirect 54**	3	3	3	**9**	**Indirect 24**	2	4	0	**6**	0.367
End organ disease		0	0	0	**0**		1	0	0	**1**		0	0	0	**0**	0.895
Sub-arachnoid hemorrhage		0	0	0	**0**		0	0	1	**1**		1	0	0	**1**	0.397
Malignancy		3	0	0	**3**		1	0	0	**1**		0	1	0	**1**	0.107
ART complication		0	0	0	**0**		2	0	0	**2**		0	0	0	**0**	0.852
Tuberculosis		7	0	1	**8**		15	0	*0*	**15**		**2**	0	0	**2**	0.011
Pneumonia sepsis		5	0	1	**6**		18	0	1	**19**		9	0	2	**11**	0.646
Meningitis encephalitis		1	0	0	**1**		4	0	1	**5**		2	0	0	**2**	0.885
Other		0	0	0	**0**		0	1	0	**1**		0	0	1	**1**	0.397
Unknown	**Unknown6**	2	0	4	**6**	**Unknown 5**	2	3	0	**5**	**Unknown 0**	0	0	0	**0**	0.002

*P value compares proportion of all deaths (HIV positive, negative and unknown) from each condition in the 3 time periods using a Chi-square test for trend. ‘Other’ deaths in 2010–2012 were pregnancy-induced aplastic anaemia and toxin ingestion, and in 2005–2009 were hypoperfusion and systemic lupus erythematosus.

In the last two years of the audit (data not available for the previous years), 96% of all women giving birth at CMJAH had attended ANC (18,752/19,524), whereas among women who had died at the facility over the 10 year period, only 61.7% had attended ANC “S1 Table” (124/201; *P*<0.001). ANC attendance was 77.4% among the HIV negative women who died (41/53), 64.8% in HIV positive women (70/108) and 32.5% in women with an unknown HIV status (13/40; *P*<0.001). Of the women who died, the proportion referred from another facility to CMJAH during labor rose over time from 34.8% in period one, to 50.0% in period two and up to 58.5% in period three (*P* = 0.02). Similarly, markedly more women who died in period three had been in a critical condition upon admission to hospital (68.9%, 42/61), with the levels highest among HIV-negative women. Regardless of time period, deaths occurred mostly after childbirth (72.0%, 167/232), mainly within the first week postpartum (45.5%, 80/176).

## Discussion

Over the ten years audited, maternal mortality in CMJAH declined among HIV-positive women, and a corresponding rise occurred in coverage of HIV testing and ART across the sub-district. A decline in overall maternal mortality in the third period mirrors the falling levels noted nationally since 2009[2,4] Our audit showed, however, that no reduction had taken place in mortality from direct obstetric causes at the facility. Moreover, the numbers of deaths from obstetric hemorrhage were concerning in each study period, and mortality from hemorrhage has risen elsewhere in the country.[4]

Declines in maternal deaths as a result of the expansion of ART access have also occurred in other parts of South Africa, including at another large tertiary hospital in Gauteng Province.[4,20] Although deaths in the third period of this review still remain three-fold higher among HIV-positive than negative women, this differential is half of what it was in the first period. In an audit of all maternal deaths in South Africa for the years 2008 to 2010, the relative risk of dying during pregnancy was six-fold higher for HIV-positive compared with HIV-negative women.[2] This same figure was found in a systematic review of international studies on this topic.[1] Over time, the relaxing of the criteria for initiating ART in women has meant that a larger proportion of HIV-positive women have become eligible for treatment. In 2015, treatment in South Africa was extended to all HIV-infected adults and children, irrespective of CD4 cell count. Clearly it is important to enact this policy as HIV-infected women with untreated HIV (or only brief periods of ART) clearly remain at high risk for direct obstetric deaths. Further rises in ART coverage may mean that the MMR among HIV-infected women (from both indirect and direct causes) will more closely approximate the MMR among HIV-negative women. Importantly, tuberculosis deaths in pregnant women, as with all adults, have declined with increasing ART coverage.[21] In this study, we noted a stepwise reduction over time in deaths from tuberculosis, as well as from HIV-associated malignancy.

Several notable changes in service delivery took place across the timespan of the audit. By 2012, 19 of every 20 women had a known HIV status, a prerequisite for ART initiation, whereas fewer than half had been tested in 2003. Decentralization of ART to primary health facilities was also key to the expansion of ART coverage, reducing referrals to the tertiary center for ART initiation and bringing HIV care closer to patients. Moreover, the present practice of initiating ART on the same day as HIV testing even further simplifies care. Finally, it is encouraging that both stavudine and nevirapine, which contributed to maternal mortality in this study, are no longer prescribed to pregnant women.

Non-attendance or limited attendance at antenatal clinics is perhaps now the principal avoidable factor for maternal deaths. In our audit, 40% of women who died had not attended care. The most common barriers to accessing antenatal care are: high indirect costs to pregnant women of attending care,[22,23] antenatal services being at odds with local cultural contexts, overloading of health services with long waiting times, and tainting of health services by previous experiences of being turned away, or abused, by health workers.[24–26].Postpartum care also remains important: nearly three quarters of deaths occurred in the week after childbirth in this study, and in the country as a whole, a high proportion of women who access PMTCT services during pregnancy are not retained postpartum.[8,27]. Moreover, future reductions in the MMR at the facility are contingent upon improvements in quality of care in primary and secondary level facilities, aiming to decrease the number of women who are referred to the hospital already in labor and in a critical condition.

Several factors relating to human resources may also have contributed to the altered patterns described above. Labor disputes appear to have impacted on service delivery,[13] most evident in the substantially lower HIV testing rates in 2010. Furthermore, frequent changes took place in guidelines for drug regimens and indications for ART during the review period. Delays in implementing guideline changes are common place. Orientation of staff to new guidelines requires time, possibly at the expense of training to improve the overall quality of obstetric care. Hopefully, the current guidelines, which include lifelong ART for all pregnant women regardless of CD4 cell count, will hold for some years, thereby allowing future efforts to build human resource capacity to be centered around initiatives, such as Essential Steps in the Management of Obstetric Emergencies (ESMOE). These are underway in the country, but require additional state and external support.[28]

The study has several limitations. It presents data collected from multiple sources and primarily for purposes other than research. The absence of a unique patient identifier means it was not possible to determine the extent to which women in each database overlap. But, we believe that data collection systems for ART initiation births and deaths are robust and it would be rare to have missing data for these events. During periods of labor dispute, however, the quality of the data may have declined, as the remaining or replacement staff prioritized service delivery over record keeping. Also, some data, such as for number of births, was only available as annual data, not for individual months, meaning that the different periods of ART provision could not be fully demarcated and the implementation of guidelines. A reduction in the number of deaths from unknown causes does, however, suggest a rise in data quality at the facility. Also, post-mortems were seldom done, raising chances of death misclassification. The dates provided for changes to the guidelines are when facilities were authorized to prescribe at the higher CD4 cell counts. Every effort was made prior to the release of the guidelines to train staff and ensure rapid implementation of the guidelines, however, some facilities may have had a delay in implementing the changes fully, making it difficult to definitively draw distinctions between the time periods. Further, there are difficulties with comparing facility-based MMR with other maternal mortality estimates, especially those from lower levels of care.[18,29] While the hospital is well-resourced and situated within the wealthiest region of southern Africa, it is a referral center and complicated pregnancies constitute a disproportionate number of deliveries, with concomitant higher mortality rates. Changes in patterns of referral to the facility also limit direct comparisons over time. The proportion of high- and low-risk women delivering at tertiary-level facilities may fluctuate over time, possibly confounding comparisons of mortality rates over time. Also, a reduction in admissions for women with severe HIV-related morbidity may have made space available for women with high-risk pregnancies, who might previously have been managed at lower-level facilities. Data on age and parity, for example, were not available on all women at the facility, which would have allowed us to adjust for changes in the study population over time. Absence of these data is a major study limitation.

## Conclusion

This study, reporting data from a tertiary-level facility, shows a link between the expansion of ART services to pregnant women and decreasing maternal mortality. Changes in referral and care patterns over time, however, diminish the ability to draw causal inferences. As access to ART services across South Africa continues to increase, this should lead to further declines in mortality.

Despite the inherent limitations of the data, the audit provides actionable information through identifying areas requiring urgent attention, including actively addressing barriers to utilization of antenatal care, and improving management of hemorrhage and other obstetric conditions at the facility and lower levels of care. The study also provides evidence to support ongoing activities in well-performing areas, such as the continued expansion of HIV testing and ART services. Ultimately, reductions in the overall MMR are contingent upon improvements in the management of obstetric emergencies, and on further rises in ART coverage and a consequent narrowing of the remaining differentials in risk for maternal death in women with and without HIV.

## Supporting Information

S1 Table Characteristics of all women who died at Charlotte Maxeke Johannesburg Academic Hospital, in the three study periods, by HIV status. Data are presented for all women and for HIV sub-group. Denominators vary due to missing data. Kruskal-Wallis equality of populations rank test. ^^^Chi-square test for differences between all HIV positive and negative women. ^&^Chi-square test for trend over 3 periods. *p<0.005,P = 0.05–0.10
(DOCX)
