# Supplementary material for: Increased Access to Antiretroviral Therapy Is Associated with Reduced Maternal Mortality in Johannesburg, South Africa: An Audit from 2003-2012
Source: PLoS One. 2016 Dec 29;11(12):e0168199. doi: 10.1371/journal.pone.0168199 (PMC5199074; doi:10.1371/journal.pone.0168199)
Supplement: S1 Table — Data are presented for all women and for HIV sub-group. Denominators vary due to missing data. Kruskal-Wallis equality of populations rank test. ^Chi-square test for differences between all HIV positive and negative women. &Chi-square test for trend over 3 periods. *p<0.005,P = 0.05–0.10 (DOCX) [file pone.0168199.s001.docx]

**Supplementary Table 1: Characteristics of all women who died at Charlotte Maxeke Johannesburg Academic Hospital, in the three study periods, by HIV status**

| **Variable**  row % (n/N) or  median (IQR), n | **All maternal deaths  2003-2012^** | **Maternal deaths  Period 1  2003-2004** | **Maternal deaths**  **Period 2  2005-2009** | **Maternal deaths Period 3  2010-2012** | ***P^&^*** | ***P***^$^ |
| --- | --- | --- | --- | --- | --- | --- |
| **HIV status all women** | 68.4 (121/177) | 82.1 (23/28) | 69.5 (66/95) | 59.3 (32/54) | 0.03 | 0.10 |
| **Known HIV status** | 76.3 (177/232) | 62.2 (28/45) | 78.5 (95/121) | 81.2 (54/66) | 0.03 | 0.04 |
| **Median maternal age**  All women  HIV positive  HIV negative | 29 (25-33.5), 232  29 (25-32), 121  30 (25.5-35), 56 | 28 (23-32), 45  28 (23-32), 23  30 (24-36), 5 | 29 (24-34),121  29 (25-34), 66  30 (24-35), 29 | 29.5 (26-34), 66  30.5 (27-32.5),32  30 (26-35), 22 | 0.18  0.34  0.75 | - |
| **Median gravidity**  All women  HIV positive  HIV negative | 2 (2-3), 221  2 (2-3), 119  3 (2-4), 55\| | 2 (1-3), 42  2 (1-3), 22  3 (2-3),5 | 2 (2-3), 115  2 (2-3), 65  3 (2-4), 28 | 2.5 (2-3), 64  2 (2-3), 32  3 (2-3), 22 | 0.17  0.26  0.95 | - |
| **Attended antenatal care**  All women  HIV positive  HIV negative | 61.7 (124/201)  64.8\| (70/108)  77.4 (41/53) | 51.3 (20/39)  54.6 (12/22)  80.0 (4/5) | 65.1 (67/103)  65.5 (38/58)  74.1 (20/27) | 62.7 (37/59)  71.4 (20/28)  81.0 (17/21) | 0.33  0.22  0.75 | 0.32  0.46  0.85 |
| **Median gestation at death**  All women  HIV positive  HIV negative | 31 (26-36), 197  30 (26-34), 105*  34 (26-38), 54 | 31.5 (24-33), 38  31 (25-32.5), 20  32 (28-33), 5 | 32 (28-36), 101  31 (28-35), 57  36 (31-38), 27 | 28 (24-34), 58  28 (25-33.5), 28  31 (26-35), 22 | 0.03  0.08  0.08 | - |
| **Referred CMJAH during labor**  All women  HIV positive  HIV negative | 49.8 (115/231)  47.1 (57/121)  48.2 (27/56) | 35.6 (16/45)  30.4 (7/23)  20.0 (1/5) | 50.4 (61/121)  51.5 (34/66)  48.2 (14/29) | 58.5 (38/65)  50.0 (16/32)  54.5 (12/22) | 0.02  0.20  0.23 | 0.06  0.21  0.38 |
| **Critical condition on admission**  All women  HIV positive  HIV negative | 38.6 (83/215)  33.0* (36/109)  49.1 (27/55) | 2.3 (1/44)  0.0 (0/23)  0.0 (0/4) | 36.4 (40/110)  32.8 (19/58) 34.5 (10/29) | 68.9 (42/61)  60.7 (17/28)  77.3 (17/22) | <0.001  <0.001  <0.001 | <0.001  <0.001  0.001 |
| **Death occurred postpartum**  All women  HIV positive  HIV negative | 72.0 (167/232)  76.9 (93/121)  73.2 (41/56) | 77.8 (35/45)  82.6 (19/23)  80.0 (4/5) | 72.7 (88/121)  80.3 (53/66)  75.9 (22/29) | 66.7 (44/66)  65.6 (21/32)  68.2 (15/22) | 0.19  0.12  0.49 | 0.43  0.21  0.78 |
| **Caesarian section delivery**  All women  HIV positive  HIV negative | 48.0 (85/177)  40.4* (38/94)  60.4 (29/48) | 38.2 (13/34)  27.8 (5/18)  40.0 (2/5) | 55.6 (50/90)  45.1 (23/51)  75.0 (18/24) | 41.5 (22/53)  40.0 (10/25)  47.4 (9/19) | 0.98  0.50  0.51 | 0.12  0.44  0.12 |
| **WHO HIV Clinical Stage 4**  HIV positive | 58.1 (54/93) | 53.3 (8/15) | 61.1 (33/54) | 54.2 (13/24) | 0.94 | 0.78 |
| **CD4 cell count below 50**  HIV positive | 42.2 (38/90) | 25.0 (3/12) | 43.4 (23/53) | 48.0 (12/25) | 0.23 | 0.40 |

Data are presented for all women and for HIV sub-groups. Denominators vary due to missing data.. ^Chi-square test for differences between all HIV positive and negative women. ^&^Chi-square test for trend over 3 periods or Kruskal-Wallis equality-of-populations rank test ^$^Chi-squared test of homogeneity of odds. * P<0.005, |P=0.05-0.10
